# Thirty years of SET/TAF1β/I2PP2A: from the identification of the biological functions to its implications in cancer and Alzheimer’s disease

**DOI:** 10.1042/BSR20221280

**Published:** 2022-11-21

**Authors:** Antonella Di Mambro, Maria Teresa Esposito

**Affiliations:** The Centre for Integrated Research in Life and Health Sciences, School of Health and Life Science, University of Roehampton, London, U.K.

**Keywords:** Alzheimer disease, cancer, I2PP2A, leukaemia, PP2A, SET

## Abstract

The gene encoding for the protein SE translocation (SET) was identified for the first time 30 years ago as part of a chromosomal translocation in a patient affected by leukemia. Since then, accumulating evidence have linked overexpression of SET, aberrant SET splicing, and cellular localization to cancer progression and development of neurodegenerative tauopathies such as Alzheimer’s disease. Molecular biology tools, such as targeted genetic deletion, and pharmacological approaches based on SET antagonist peptides, have contributed to unveil the molecular functions of SET and its implications in human pathogenesis. In this review, we provide an overview of the functions of SET as inhibitor of histone and non-histone protein acetylation and as a potent endogenous inhibitor of serine–threonine phosphatase PP2A. We discuss the role of SET in multiple cellular processes, including chromatin remodelling and gene transcription, DNA repair, oxidative stress, cell cycle, apoptosis cell migration and differentiation. We review the molecular mechanisms linking SET dysregulation to tumorigenesis and discuss how SET commits neurons to progressive cell death in Alzheimer’s disease, highlighting the rationale of exploiting SET as a therapeutic target for cancer and neurodegenerative tauopathies.

## Introduction

**Figure 1:**
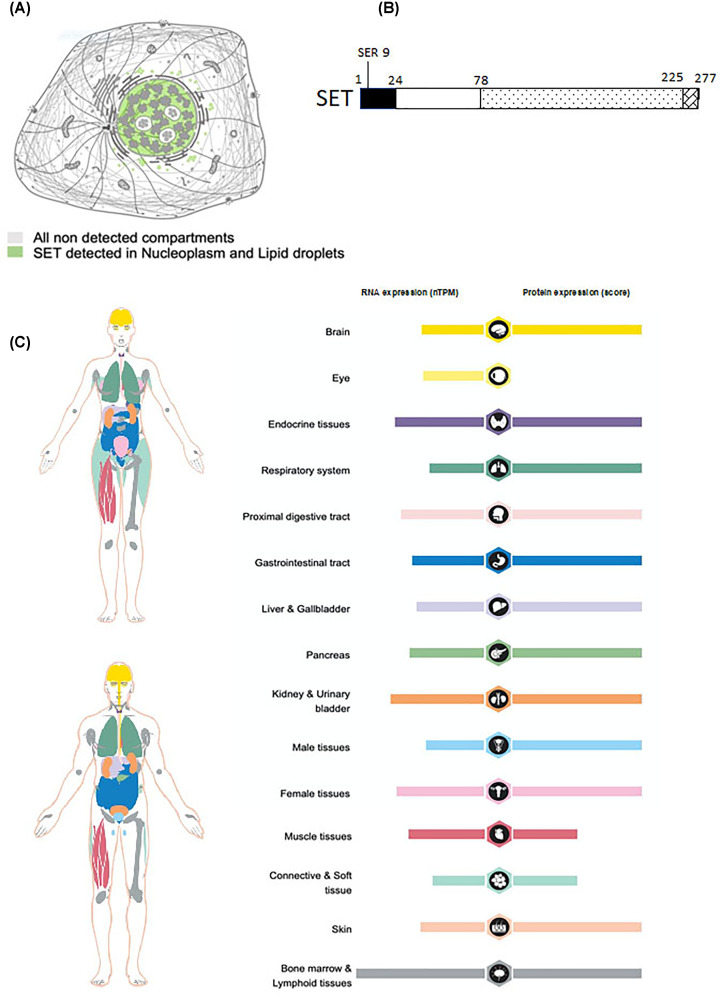
Overview of SET localization, structure and expression in tissues (**A**) Subcellular localization of SET. SET is mostly localized within the nucleoplasm. In addition, part of this protein has been found localized in liquid droplets within the cytoplasmic compartment. Image credit: Human Protein Atlas, www.proteinatlas.org. Image available at the following URL: v21.proteinatlas.org/ENSG00000119335-SET/subcellular. (**B**) Diagram of SET. Structurally, SET consists of several domains, each with specific functions: a N-terminal region (aa 1–24), a backbone helix (aa 25–78), an earmuff domain (aa 79–225) and a long acidic tail in the C-terminal region mediating the interaction with specific proteins such as p53, Ku70, Foxo1, KLF5. (**C**) Expression of SET in adult tissue cells. RNA expression summary shows the consensus data based on normalized expression (nTPM) values from RNA-seq data generated from Human protein Atlas and from the Genotype-Tissue Expression project. Protein expression scores are based on a best estimate of the ‘true’ protein expression from a knowledge-based annotation (details at : v21.proteinatlas.org/about/assays+annotation **-** ihk). Each bar represents the highest expression score found in a particular group of tissues. Color-coding is based on the tissue groups, each consisting of tissues with functional features in common. Image credit: Human Protein Atlas, www.proteinatlas.org. Image available at the following URL:v21.proteinatlas.org/ENSG00000119335-SET/tissue

The gene patient SE translocation (SET), on locus 9q34, was identified for the first time 30 years ago as part of a chromosomal translocation with the gene *CAN* (now known as *NUP214*) in a patient affected by acute undifferentiated leukaemia [1,2]. Also known as inhibitor 2 of PP2A (I_2_PP2A), template activating factor-Iβ (TAF-Iβ) and putative histocompatibility leukocyte antigen class II-associated protein, SET is not a member of the SET (Su(var)3-9, Enhancer-of-zeste, Trithorax) domain protein methyltransferases but a multi-tasking protein expressed in all tissues, predominantly localised in the nucleus and able shuttle to the cytosol [3] (Figure 1A). Accumulating evidence have revealed that translocation of SET in the cytosol is implicated in neurodegenerative tauopathies such as Alzheimer’s disease (AD) and amyotrophic lateral sclerosis (ALS) [4–6]. Overexpression of SET has been reported in several solid tumors and hematological malignancies [7–12] and SET expression has been functionally linked to poor prognosis, progression of the neoplastic disease and development of resistance to treatment [7,11,13,14]. Therefore, the biological characterization of SET has attracted significant attention in the scientific community and progress has been made in understanding the physiological and pathological functions of this protein. SET is a subunit of the inhibitor of acetyltransferases complex (INHAT) that modulates histone acetylation and gene transcription [15]. In addition, it is a potent endogenous inhibitor of tumor suppressor serine-threonine phosphatase PP2A [16,17], whereby SET impairs the activity of PP2A toward its targets, including several kinases which mediate cancer cells’ survival [12,18,19] and the protein tau, which is key in the development of AD and other tauopathies [20]. These mechanisms provide the molecular link between SET, inhibition of PP2A, cancer progression and AD.

## Molecular structure and sub-cellular localization of SET

SET is transcribed from a gene on chromosome 9q34, which gives rise to four transcript variants: variant α (also known as TAF1α 2863 bp; 290 aa; 41 KDa); variant β (SET 2936 bp; 277 aa; 39 KDa); variant γ (2638 bp, 265 aa); variant δ (2562 bp, 268 aa) [1,3]. These forms differ in the first exons and they have in common the sequences from exon 5 to exon 11. Structurally, SET consists of several domains, each with specific functions (Figure 1B): a N-terminal region (aa 1–24), a backbone helix (aa 25–78) and an earmuff domain (aa 79–225), that together make the nucleosome assembly protein (NAP) domain (aa 25–225), and a long acidic tail in the C-terminal region (aa 226–277) [15,21,22]. The N-terminal region (aa 1–76) is essential for SET dimerization. In the dimer, the two backbone helices interact hydrophobically in an antiparallel manner. Several residues in the backbone helix are essential to contribute to dimer formation [22]. The backbone helix is also responsible for nucleosome assembly. The SET earmuff domain binds both core histones, preferentially histone H3 and H4 [23–25], and dsDNA and it is involved in histone chaperone activity, whereas the acidic tail of the C-terminus mediates the interaction with specific proteins such as p53, Ku70, FOXO1, KLF5, as further described below [26–28]. The interaction between SET and PP2A involves several SET domains encompassing the residues 25**–**119 near the N-terminus and the Val92 [16,21,29]. Moreover, phosphorylation of SET on Serine 9 has been shown to increase the affinity between SET and PP2A and to mediate the accumulation of SET in the cytoplasm [30,31].

SET is ubiquitusly expressed in a variety of adult tissue cells (Figure 1C) [3] and it is also expressed during embryogenesis. In zebrafish, two paralogs, *Seta* and *Setb*, are 96% identical to human *SET*. In zebrafish embryos at the stage of 24 *hpf* (hours post fertilization), *Seta* and *Setb* are expressed in the brain, the midbrain–hindbrain boundary and the eye retina. *Seta* is also detected in the spinal cord, wehereas Setb in the tail bud. The knockdown of Set genes in zebrafish gives rise to morphants with smaller and distorted eyes; the gene expression profile of these morphants indicated a marked decrease in the expression of genes important for the sensory organ development, cell adeshion and an increase in p53 target genes [32]. The *Set* knockout mice die during embryonic development between day E11.5 and E12.5 post coitum. The embryos show cardiac edema and open neural tube, delayed growth and up-regulation of p53 and Foxo1 target genes [33]. This is consistent with the observation that SET regulates the expression of genes involved in the organogenesis of the spinal cord [34].

## Physiological roles of SET

SET is actively involved in numerous cellular processes including DNA replication, chromatin remodeling, gene transcription, DNA repair, cell migration and cell cycle regulation. Some of these effects are mediated by inhibition of histone acetylation, other by inhibition of PP2A activity or by specific interaction with protein partners. In this paragraph, we review our knowledge of the physiological roles of SET.

### Inhibition of histone and non-histone protein acetylation

SET, together with the tumor suppressor ANP32A (also known as PP32 and I1PP2A), forms the inhibitor of acetyltransferases (INHAT) complex that regulates several cellular functions by modulating histone and non histone protein acetylation [15,35].

#### Chromatin remodeling and gene transcription

**Figure 2:**
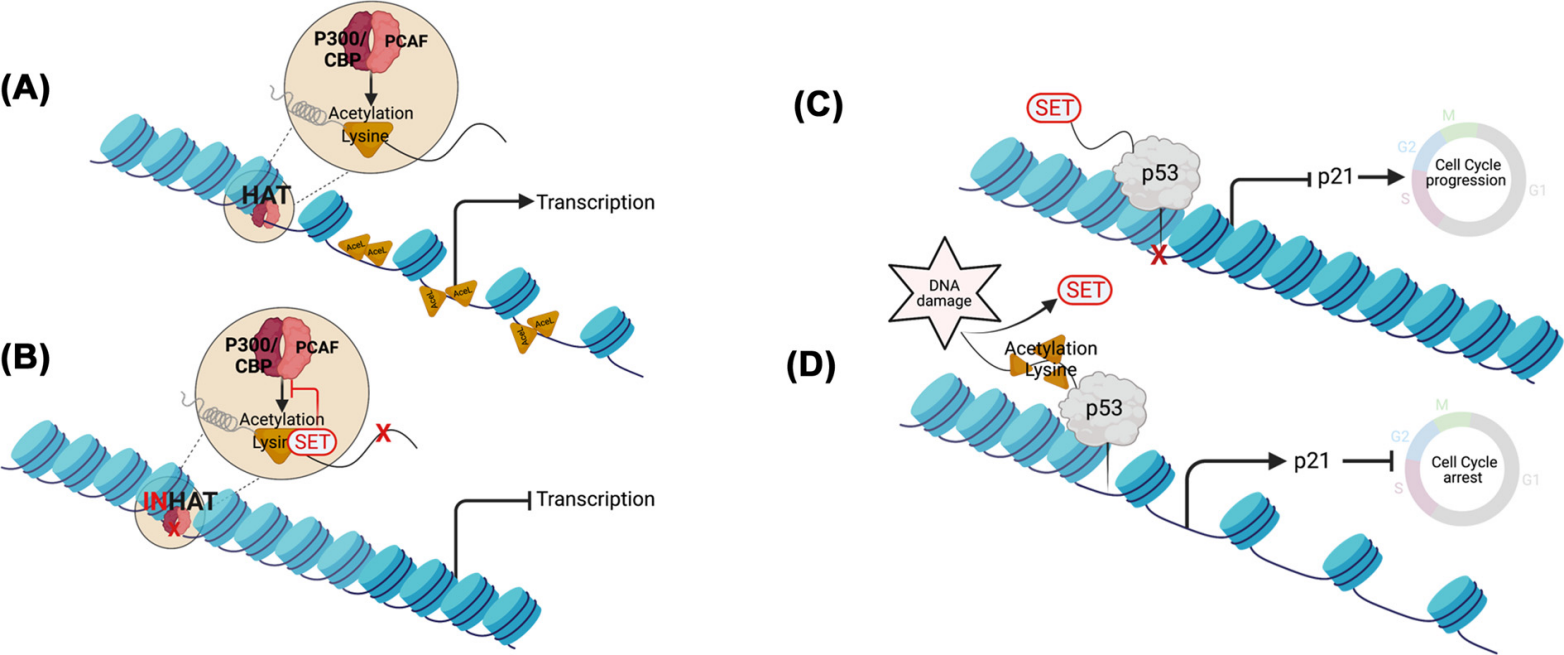
Role of SET in chromatin remodeling and gene transcription As part of the INHAT complex (inhibitor of acetyl transferase) SET acts as a transcriptional repressor by masking histones and non histone proteins such as p53, Foxo, Sp1 and KLF5 from acetylation. (**A**) The histone acetyltransferases (HAT) complex comprises enzymes responsible for histone acetylation, such as p300/CBP (CREB-binding protein) and PCAF (p300/CBP-associated factor). The acetylation of histones relaxes the chromatin prior transcription. (**B**) As part of the INHAT complex, SET prevents histones acetylation from the HAT complex with resultant transcription silencing. (**C**) SET protects non histone proteins such as p53 from acetylation. In unstressed cells, the acidic domain of SET interacts with p53, inhibiting its binding to DNA and transcriptional activity. As a consequence, cells can progress in the cell cycle. (**D**) In response to cellular stress and DNA damage, the SET–p53 interaction is disrupted and p53 is acetylated. As a result, p53 activates the transcription of downstream targets involved in cell cycle regulation such as cell cycle inhibitor p21. Image generated using Biorender.

Histone acetylation changes the overall charge on the histone tails from positive to neutral. As a result, the interaction between DNA and histone proteins is weakened, the chromatin is decondensed and the DNA becomes more accessible for transcription [36]. Acetylation marks are added by histone acetyltransferases (HATs) such as p300/CBP (CREB-binding protein) and PCAF (p300/CBP-associated factor) that can also directly acetylate transcription factors and modulate their ability to bind to gene promoters [36]. The INHAT complex binds the lysine residues at the histone amino-terminal tails occluding the histones from serving as an acetylase substrate for HATs, essential for chromatin accessibility and chromatin transcription. As part of the INHAT complex, SET binds to unacetylated and hypoacetylated histones preventing histone acetylation by p300/CBP and PCAF and repressing gene transcription (Figure 2) [15,23,24,35]. SET can also recognise, on gene promoters, H3K27me1/2/3, a repressive mark deposited by PRC2 [37]. In this way SET fosters an additional layer of transcriptional repression. Interestingly, when the serine residue S28 adjacent to lysine K27 is phosphorylated (H3S28), SET can no longer bind to the target gene promoter and H3K27 modification is changed to acetylation by HAT, switching the mark from repressive (H3K27 methylation) to activating (H3K27 acetylation) [37]. This provides evidence that post-translational modifications on histones may form part of a binary switch mechanism where repressive marks can be gradually replaced by activating marks with SET reinforcing the repressive mark. Moreover, SET binding to histones affects chromatin remodelling and histone folding. Saavedra et al. showed that by binding to H4, SET and ANP32A inhibit the acetylation on specific lysine residues [38]. When early acetylation occurs, H4 can no longer bind to HSP90. This binding is essential to ensure H4 stability and it might be important to allow H4 to pass through further post-translational modifications and marks deposition [38].

In addition to inhibiting histone acetylation, SET represses gene transcription by inhibiting the acetylation of non histone proteins such as the transcription factors p53, FOXO1, Sp1 and KLF5 [26–28,33,39]. SET interacts with the Carboxy-terminal domain (CTD) of p53, a region containing a lysine rich domain found also in other SET interactors (Table 1). SET mediates inhibition of the acetylation of this domain by p300, repressing the transcription of p53 target genes and blocking p53-mediated cell cycle arrest and apoptosis [39] (Figure 2). However, the study by Kim *et al.* left uncovered how acetylation of p53, in response to stress or DNA damaging agents, affected the interaction between SET and p53. A few years later Wang *et al.* reported that SET binds to unacetylated p53 in unstressed cells and, by doing so, it represses the transcriptional activity of p53. However, upon DNA damage, the interaction between p53 and SET diminishes, probably owing to the induction of p53 acetylation [26] (Figure 2C,D). When, in human cancer cell lines, SET is knocked down by RNA interference, the expression of p53 target genes *CDKN1A* and *PUMA* encoding, respectively, for the cell cycle inhibitor p21 and p53 upregulated modulator of apoptosis, is up-regulated, without changes in p53 protein stability. Interestingly, these effects are abrogated in isogenic cells lines knockout (KO) for *TP53* [26]. Similar results were obtained by analyzing the gene expression in the tissues isolated from *Set* KO mouse embryos, indicating that loss of *Set* led to increased p53 transcriptional acitivity towards *Cdkn1a* and *Puma* [33]. Furthermore, ChIP analyses showed that SET suppressed p300-dependent acetylation of H3K18 and H3K27 on the promoters of *CDKN1A* and *PUMA* indicating that SET exerts a dual inhibitory effect on p53 transcriptional activity by binding to p53 and protecting it from acetylation and also by suppressing the acetylation of its target genes' promoters [26] (Figure 2C,D).

**Table 1:** List of SET interacting proteins and relative function

Interacting protein	Functions and reference
AEP	Induction of apoptosis [122]
ApoE	SET inhibition [11]
Cyclin B-CDK1	Regulation of cell cycle [62]
Cytochrome c	Inhibition of SET [137]
E-CDK2-p21 complex	Regulation of cell cycle [61]
Estrogen Receptor (ER)	Inhibition of transcription of downstream targets [41,42]
FOXO1	Inhibition of transcription of downstream targets [26]
Glucocorticoid receptor (GR)	Inhibition of transcription of downstream targets [41]
Granzyme A	Induction of apoptosis [65,66]
Histone H1	Regulation of transcription [42]
Histone H2A	Regulation of transcription [138]
Histone H2B	Regulation of transcription[25,138]
Histone H3	Regulation of transcription [15,23–25]
Histone H4	Regulation of transcription [38]
hnRNPA2	Cytosolic retention of SET [97,102]
Jcasp fragment	Induction of neuronal death [64]
KAP1	Repression of DNA damage repair by HR [49]
KLF5	Inhibition of transcription of downstream targets [27]
KMT2A	Transcription activation of downstream target genes [43,79]
Ku70	Prevention of DNA damage repair by NHEJ [48]
PIKE-L	Cytosolic retention of SET [122]
PP2A	Inhibition of PP2A activity [16,21,52]
p21Cip1	Inhibition p21/Regulation of cell cycle [61]
p53	Inhibition of transcription of downstream targets [26,39]
Rac1	Regulation of cell migration [67]
Retinoic acid receptor (RAR)	Inhibition of transcription of downstream targets [41,42]
SETBP1	Cytosolic retention of SET [97,101]
SP1	Inhibition of transcription of downstream targets [28]

SET interacts also with the transcription factor FOXO1, that plays a key role in the activation of the expression of various target genes involved in metabolism, cell cycle, cell death, DNA repair and oxidative stress response (Table 1). FOXO1 is acetylated by p300/CBP and PCAF in response to oxidative stress and its acetylation decreases its transcriptional activity through attenuation of its DNA binding ability. Similar to the studies concerning the interaction between SET and p53, Wang *et al.* showed that SET can interact only with unacetylated FOXO1 and, by doing so, it represses the transcriptional activity of FOXO1 [26]. Indeed, in the tissues isolated from *Set* KO mouse embryos, *Foxo1* target genes *Cdkn1b*, encoding for the cell cycle inhibitor p27, *Ccng2*, encoding for Cyclin G2 and *Dr5*, encodying for Death Receptor 5 were found up-regulated, suggesting that SET exerts an inhibitory effect on FOXO1 mediated transcription [33]. These data are in contrast with those published by Chae* et al.* that indicate that SET overexpression results in the up-regulation of *CDKN1B* [40]. A potential explanation for these contrasting results might lay in the models used for the studies. Indeed, Chae *et al.* used a human cancer cell line knocked out for *Tp53* [40]. Deletion of *Tp53* partially rescues the phenotype of *Set* KO mice and therefore it might mask the true effect of SET on FOXO1 transcriptional activity.

SET has also been shown to interact with two members of the Sp1/Kruppel-like factor family of zinc finger transcription factors, KLF5 and SP1 [27,28] (Table 1). The interaction between SET and KLF5 and SET and SP1 leads to inhibition of the binding of these transcription factors to their target genes’ promoters [27,28]. In addition, SET has been shown to mask the acetylation site of KLF5 to p300; this also contributes to impair KLF5-DNA binding and transactivation activities [28].

SET binds to the DNA binding domain of the glucocorticoid receptor (GR), the retinoic acid receptor (RAR) and the Estrogen Receptor (ER) and inhibits the transcriptional activity of these nuclear receptors [41]. The interaction between SET and the GR responsive elements (GREs) on target gene promoters has profound implications on the resistance of *SET-NUP214* T-cell Acute Lymphoblastic Leukemia (T-ALL) to corticosteroids, possibly due to inappropriate transcriptional regulation of GR target genes, as described in the following paragraphs [41]. Furthermore, the SET-mediated inhibition of the transcriptional activity of the RAR and ER is also consistent with the findings published by Kato* et al.* that reported that in HeLa cells where *SET* was knocked-down by RNA interference, there was up-regulation of several ER-responsive genes, including *TPD52L1*, *TFAP2C*, *DNAJC12* and *TFF1* [42].

In contrast to these repressive roles, SET stimulates the transcription of a sub-set of genes, including the cytochrome *P450c17* [34], *HOXA* [43] and *KAI*1 [44] and it is involved in the stimulation of the CBP -mediated transcription [45]. This stimulatory effect might be due to interaction with other epigenetic regulators and it might have an important role in the development of certain diseases such as hematological malignancies as described below.

#### Chromatin remodeling—DNA methylation

Two studies have investigated the effect of SET in DNA methylation, a chromatin modification associated to gene silencing. In a study conducted by Cervoni *et al.* overexpression of *SET* in HEK-293 cell line blocked demethylation of ectopically methylated DNA resulting in gene silencing [46]. With a similar approach, Almeida *et al.* compared the methylation profiles of isogenic cell lines where *SET* was overexpressed and they found that *SET*-overexpressing cells showed loss of DNA methylation in the promoters of several tumour suppressor genes, in contradiction with the previous study published by Cervoni *et al.* [47]. As promoter hypomethylation is usually associated with increased chromatin accessibility and gene transcription, the authors checked the gene expression profile in *SET* overexpressing cells and they found that in these cells, whereas several gene promoters were hypomethylated, the corresponding genes were not overexpressed, but instead down-regulated, suggesting that SET regulates gene expression through a mechanism independent of DNA methylation. By using pharmacological inhibitors of histone deacetylases (HDAC) the authors demonstrated that the main mechanism of SET-mediated gene silencing involved histone hypoacetylation and chromatin compacting in *SET*-overexpressing cells [47].

#### DNA repair

SET has inhibitory effects on DNA damage repair. SET interacts with Ku70, that together with Ku80 make up the Ku heterodimer, a fundamental molecular complex in non homologous end joining (NHEJ) DNA repair (Table 1). UV-induced DNA damage disrupts the interaction between SET and Ku70 and it releases the heterodimer Ku70/Ku80, which is then recruited to DNA double strand breaks (DSB) sites to start the NHEJ-mediated DNA repair pathway. Overexpression of SET inhibits the recruitment of Ku70/80 to DNA damage sites by inhibiting p300/CBP and PCAF-mediated acetylation of Ku70. In this way, SET overexpression prevents repair of damaged DNA by NHEJ, and it also contributes to abnormal cellular proliferation [48]. In addition, SET plays also a role in Homologous Recombination (HR) DNA repair. In eukaryotic cells, DNA repair generally occurs in the context of highly structured chromatin and, as a result, the cell has evolved mechanisms to open the chromatin structure and facilitate repair, such as histone modifications and recruitment of other chromatin proteins, such as the Kruppel-associated box (KRAB)-associated co-repressor KAP1. Upon binding of SET to KAP1, KAP1 recruits the methyltransferase SETDB1 for trimethylation of histone H3 at Lys-9 (H3K9me3) and heterochromatin protein 1 (HP1). The SET/KAP1/HP1/H3K9me3 association on the DSBs leads to chromatin retention and creates a repressive environment for DNA repair. In this way, SET prevents repair of damaged DNA by HR [49]. Moreover, overexpression of SET fails to induce up-regulation of *ATM*, *BRCA1* and *CHK2* and recruitment of BRCA2 to DNA damage foci in head and neck squamous cell carcinoma (HNSCC) lines upon cisplatin treatment, suggesting another important inhibitory role of SET on DNA damage repair [50]. Recent evidence suggests a role for SET-mediated PP2A inactivation in DNA mismatch repair (MMR), a pathway that corrects errors associated with DNA replication and recombination that otherwise would lead to microsatellite instability (MSI) [51]. Yen *et al.* reported high level of expression of SET and CIP2A, another endogenous inhibitor of PP2A, but decreased levels of PPP2R1A (a structural subunit of PP2A) in colorectal cancers and endometrial cancers displaying MSI, a genetic hallmark of MMR defects [51]. By using conditional genetic deletion of *PPP2R1A* in mouse intestinal tumors, they demonstrated that PP2A inactivation induces epigenetic silencing of MLH1 and MMR defects, recapitulating the observations found in the human samples [51].

#### Oxidative stress

SET is involved in the response to oxidative stress by inhibiting the expression of aldehyde dehydrogenase 2 (ALDH2) and Glutathione S-transferase pi1 (GSTP1), two important detoxifying enzymes. SET down-regulates *ALDH2* and *GSTP1* gene expression by promoting histone hypoacetylation [50]. This might contribute to tumorigenesis in the presence of carcinogenic/toxic agents.

### Inhibition of phosphatase PP2A activity

SET was identified as potent non competitive inhibitor of phosphatase PP2A in 1996 [16] (Table 1). Both SET (TAF1β) and TAF1α are able to inhibit the activity of PP2A [21]. One of the first *in vivo* proof of the activity of SET as PP2A inhibitor was provided in 1999. Al Murrani *et al*. induced transient expression of SET in HEK-293 cells and they observed an increase in the DNA binding of the proto-oncogene c-Jun, that together with Fos makes up the transcriptional activator protein-1 (AP1). In contrast, expression of the catalytic subunit of PP2A (PP2A-C) markedly decreased the DNA binding of c-Jun. This effect was dependent on changes in the phosphorylation of c-Jun at Serine 63, indicating that SET elicits effects that are consistent with its acting as an inhibitor of PP2A in intact cells [52]. The mechanisms by which SET inhibits PP2A were further elucidated years later when SET was proven to bind *in vivo* to PP2A-C [29] and when it was shown that ceramide might prevent the inhibitory effects of SET on PP2A activity. Indeed, by using site-directed mutagenesis, Mukhopadhyay *et al.*, established SET as ceramide binding protein with stereoisomer and fatty acid chain length specificity (such as d-e-B-C6-Cer and as -C18-Cer). Interestingly, in response to high concentrations of C18-Pyr-Cer, the affinity between SET and PP2A decreased in a dose-dependent manner. Consequently, the inhibitory effect generated by SET on PP2A activity upon treatment with ceramide was lower than the SET inhibitory effect observed in untreated controls (no ceramide) [17]. This evidence suggested that the use of ceramide or ceramide-derivate molecules could protect PP2A from inhibition by SET and, therefore, it might represent a new therapeutic strategy for cancer treatment. In this regard FTY720 (Fingolimod, Gilenya™, Novartis Pharma), or 2-acetyl-4-tetrahydroxyimidazole, a synthetic sphingosine analog approved by the U.S. Food and Drug Administration (FDA) for the treatment of multiple sclerosis, has been widely studied as a PP2A activator [18,53,54]. Because of its structural similarity to sphingosine, FTY720 binds the lipid-binding pocket of SET which has high affinity for D-e -ceramide and compounds structurally similar to ceramide/sphingosine. As a consequence, FTY720 sequesters SET, which is released from the catalytic subunit of PP2A, leading to PP2A reactivation and tumour suppression [55–60]. As PP2A is a major tumor suppressor regulating several signaling pathways [18,19,54], the inhibition of PP2A by SET has an overall oncogenic effect as it can activate oncogenic signaling such as AKT, ERK1/2, c-MYC and GSK3-β to promote the survival and progression of cancer cells, as we describe in the following paragraphs.

### Other effects

SET has also been reported to mediate other cellular effects. Some of these effects are mediated by inhibition of histone acetylation, other by inhibition of PP2A activity and specific interaction with protein partners.

#### Cell cycle

SET is involved in the control of G1/S and G2/M cell cycle transition. Almeida *et al.* showed that whereas HEK293 cells exposed to cytotoxic drug cisplatin accumulate in G0/G1 and S phase, HEK293 cells overexpressing *SET* accumulate in G2/M, indicating that overexpression of SET interferes with the response to cisplatin by abrogating cell-cycle arrest in G0/G1 [50]. This effect might be explained by the interaction between SET and the cyclin-dependent kinase (CDK) inhibitor p21CIP1. SET binds and inhibits p21CIP1, overriding the inhibitory effect of p21CIP1 on CDK2/cyclin E and acting as positive regulator of G1/S transition [61] (Table 1). When SET binds the cyclin E-CDK2-p21 complex, it induces a conformational change in the proteins involved, overriding the inhibition of CDK2 activity by p21, allowing G1/S transition [61]. In addition to its role as positive regulator of G1/S transition, SET might also regulate the G2/M transition. Overexpression of SET in two different cellular types, COS and HCT116, induced the inhibition of cell cycle progression at G2/M transition by inhibiting cyclin B-CDK1 activity [62]. Interestingly, the inhibitory effect of SET and p21CIP1 was additive, suggesting that they might cooperate in regulating cyclin B-CDK1 activity under specific circumstances, as for instance after DNA damage, as previously demonstrated for Gadd45. In this context, SET acts as a negative regulator of G2-M transition. These data reveal that SET plays a dual role in the cell cycle as a positive regulator of G1/S and as a negative regulator of mitosis entry. The fine molecular details of the mechanism implicated in the regulation of cell cycle by SET are still unknown. SET interacts specifically with B-type cyclins, although the functional significance of this interaction has not been elucidated. Some of these effects might be mediated by the ability of SET to regulate protein acetylation. Interestingly, SET binds to two specific domains of p21CIP1 located at the C-terminus that mediate the interaction between p21CIP1 and several proteins including proliferating cell nuclear antigen (PCNA), the E7 oncoprotein of the human papilloma virus, Gadd45, c-MYC and calmodulin, among others. It is not clear whether SET competes with these other p21CIP1-binding proteins for the association with p21CIP1. As SET is also a potent regulator of the activity of phosphatase PP2A (discussed above) it might regulate the cell cycle also by controlling the phosphorylation of nuclear proteins and CDKs. Thus, the cell cycle functional steps regulated by SET remain unclear. More recently it has been shown that SET regulates Aurora B activity during mitotic cell division by inhibition of PP2A activity [63]. Aurora B, activated by Thr232 auto phosphorylation, fine-tunes the phosphorylation of kinetochore components, to regulate kinetochore-microtubes attachment. Overexpression of SET-NUP214 in cancer cells leads to inhibition of PP2A activity, dysregulation of Aurora B and destabilization of kinetochore**-**microtubule attachment with increases chromosomal instability [63].

#### Apoptosis

The role of SET in apoptosis is context dependent. As discussed in the above paragraphs, SET can modulate the transcriptional activity of p53, establishing an antiapoptotic effect in unstimulated/unstressed cells. [26]. This effect is reversed when cells are exposed to DNA damage inducing agents [26]. An antiapoptotic effect has also been reported for SET in leukemic cells, whereby SET overexpression results in decreased caspase-dependent apoptosis via a mechanism PP2A-dependent [13].

On the contrary, SET over-expression induces neuronal apoptosis [5,64]. Madeira et al. studied the mechanisms of neuronal apoptosis accompanying Alzheimer’s disease, a neurodegenerative disease that is associated with deposition of extracellular β amyloid peptide (Aβ deposits) from the amyloid precursor protein (APP) in the brain. The group inserted several APP short fragments into live neurons to understand better the phenomenon of neuronal loss in Alzheimer’s disease and they discovered that one fragment, called Jcasp, interacting with SET, induced neuronal cell death (Table 1) [64]. Moreover, SET has been shown to mediate apoptosis induced by Granzyme B, a molecular mechanism typical of cytotoxic killer T lymphocytes (CTL) and Natural killer (NK) cells [65,66].

#### Cell migration

RhoGTPase Rac1 promotes cytoplasmic accumulation of SET, that can stimulate Rac1-mediated cell migration by translocating to the plasma membrane and activating Rac1 [67]. Conversely, reduction of SET expression inhibits the RhoGTPase Rac1-induced migration, indicating that Rac1 signaling requires membrane recruitment of SET [67]. Moreover, cytoplasmic localization of SET promotes wound healing [68]. It is interesting to note that COG112, a peptide antagonist of SET, inhibits SET association with Rac1, leading to decreased migration and invasion of cancer cells [69]. It is not clear whether this effect is dependent on the activity of SET to impair protein acetylation, PP2A inactivation, or if this is simply an effect of direct interaction between SET and Rac1.

In contrast to the these data, Sobral *et al*. showed that knockdown of *SET* in HNSCC cell line HN12 increases the expression of mesenchymal markers and the migratory and invasive abilities of this cell line both *in vitro* and *in vivo* [70].

#### Cell differentiation

One of the several functions attributed to SET is cell differentiation. Kandilci *et al.* were the first to investigate the role of SET and SET-NUP214 in hematopoietic differentiation using human promonocytic cell line (U937) expressing tetracycline-regulatable Flag-epitope tagged SET (FS) [71].

U937 cells can differentiate to dendritic cell (DC) expressing specific surface markers such as CD11b and CD86. While SET-NUP214 blocks U937 cellular differentiation reducing the expression of CD11b [71], overexpression of SET promotes U937 differentiation and acquisition of DC-like features, such as expression of CD11b and CD86, in a Ca^2+^-dependent manner [72]. Rescue experiments confirmed that upon antagonism of calmodulin (CaM) with W-7 hydrochloride, or inhibition of calmodulin-dependent kinase II (CaMKII) using the KN-93 molecule, the expression of SET-induced surface markers was blocked and SET-morphologic changes reverted, suggesting that these effects were dependent on CaMKII. Given the role of CaMKII in mediating the activation of MAPK/ERK, and that MAPK/ERK pathway contributes to the maturation of human monocyte-derived DC, Kandilci *et al*. demonstrated that in FS-U937, selective inhibition of MEK1/ERK, using the inhibitor PD98059, decreased the levels of CD11b and CD86 markers, reversing the DC-like features [72].

## SET is dysregulated in cancer

**Figure 3:**
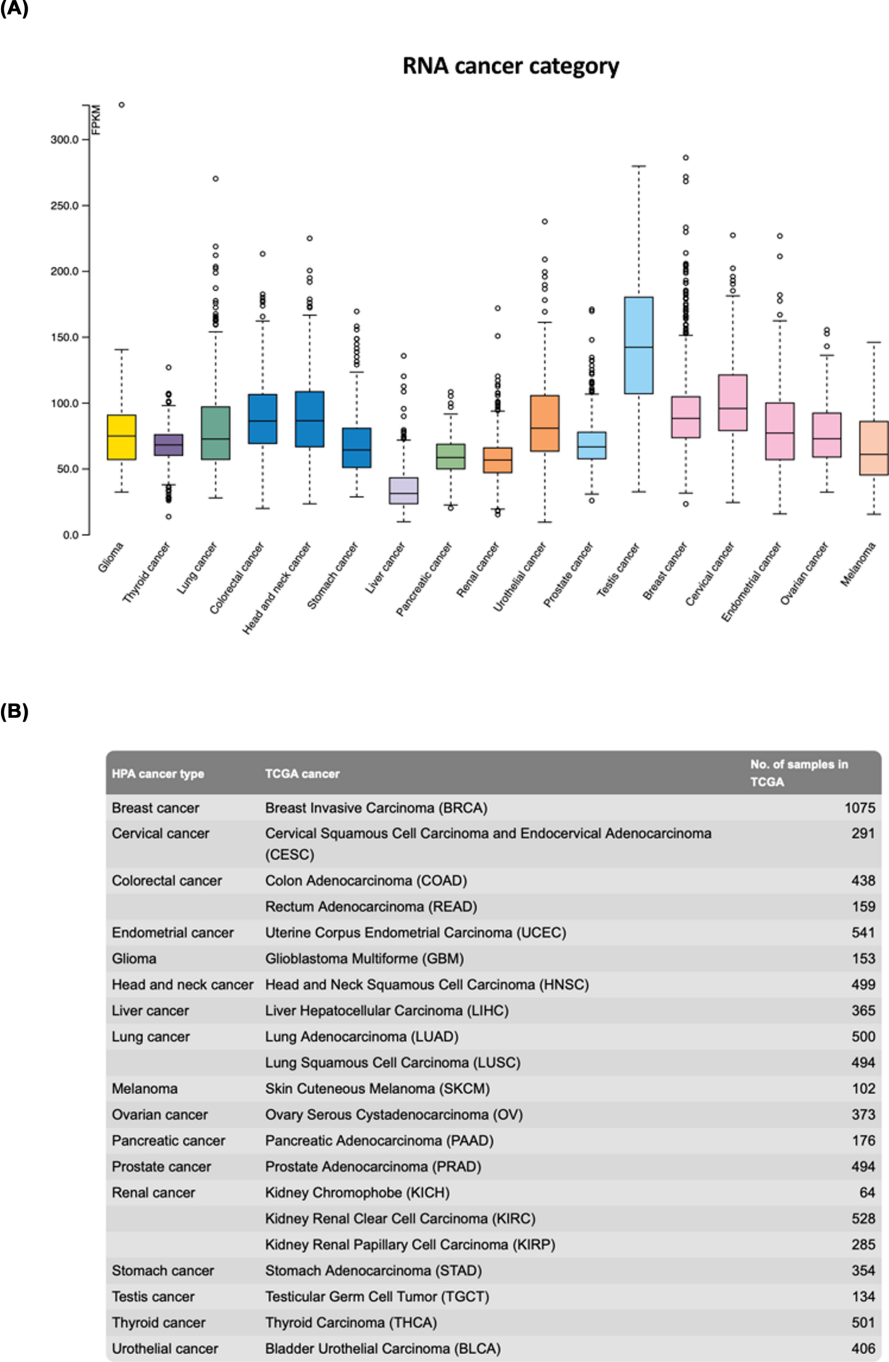
Expression of SET in cancer Analysis of mRNA expression of SET across 17 forms of cancer (21 cancer subtypes) generated by RNA-seq from The Cancer Genome Atlas. (**A**) Box plots representing normal distribution of dataset shown as median and 25th and 75th percentile. Points are displayed as outliner when above or below 1.5 times the interquartile range. Cancer types are color-coded according to the organ the cancer originates from. (**B**) Summary of cancers and subtypes of cancers included in the study, accompanied by total number of samples recruited for each subtype of cancer. Plots and Image credit: Human Protein Atlas, www.proteinatlas.org. Data available at the following URL: v21.proteinatlas.org/ENSG00000119335-SET/pathology

The malignant role of SET was first characterized in acute undifferentiated leukemia [1,2] and later associated with other forms of solid tumors, including hepatocellular carcinoma, breast cancer, lung cancer and hematological malignancies such as chronic myeloid leukemia (CML), acute myeloid leukemia (AML), T- and B-cells acute lymphocytic leukemia (T-ALL and B-ALL) and B-cells chronic lymphocytic leukemia (CLL) (Figure 3). In this paragraph, we review the mechanisms implicated in aberrant expression and activity of SET in cancer cells.

### SET as part of chromosomal translocations

In undifferentiated leukemia SET was first identified as part of chromosomal translocation *SET-NUP214* [1,2]. The fusion protein consists of almost the whole SET protein fused to the C-terminus of NUP214, leading to disorganization of SET nuclear export [73]. The fusion gene *SET-NUP214* was also reported in T-cell acute lymphoblastic leukemia (T-ALL) [1,74,75] and in one case of AML [76]. Expression of *SET-NUP214* in ALL patients highly correlates with resistance to corticosteroid treatment, possibly due to inappropriate transcriptional regulation of glucocorticoid target genes by SET-NUP214 [75]. Indeed, SET binds with the DNA-binding domain of the glucocorticoid receptor (GR) and it has been shown to inhibit the GR- transcriptional activity, but glucocorticoids can release SET and attract HATs on the glucocorticoid response elements (GRE) to drive gene transcription (Table 1) [41]. SET-NUP214, instead, was constitutively co-precipitated with GRE where it had a repressive effect on transcription, even in presence of glucocorticoids [41]. This renders cells overexpressing SET-NUP214 not responsive to glucocorticoids. Moreover, in T-ALL *SET-NUP214* promotes an elevated expression of *HOXA* genes by recruiting wild-type SET and the epigenetic regulators DOT1L and KMT2A on their promoters [77,78]. The interaction between SET and KMT2A, a gene that is frequently rearranged in acute leukemias, might suggest a role for SET in KMT2A-mediated transcription and possibly chromatin maintenance (Table 1) [43,79].

### Aberrant gene expression

SET is overexpressed in several hematological malignancies such as undifferentiated leukemia [1], T-ALL [59,74,80], BCR-ABL positive CML and B-ALL [7], B-ALL [10], B-CLL and non-Hodgkin’s lymphoma (NHL) [11], AML [13,81–83] and solid tumors, including Wilm’s tumors [84], lung tumor [57,85], metastatic colon rectal cancer [51,86,87], breast cancer [14,88,89], gastic cancer [90,91], pancreatic cancer [92], head and neck squamous carcinoma [70], endometrial cancer [51] and ovarian cancer [93] (Figure 3). Although the expression of SET has been reported in so many distinct tumors, little is known about the underlying molecular mechanisms. SET expression increases with CML disease progression and BCR-ABL1 induces SET expression [7,94]. In CLL increased levels of SET correlate with poor prognosis [11]. Studies conducted by Pippa *et al.* demonstrated that the transcription factors (TFs) complex comprised by MYC, GATA2, SP1 and RUNX1 regulates *SET* transcription. Since, apart from MYC, no DNA motifs for any of the TFs above were found on SET promoter, the evidence collected by Pippa *et al.* suggested that MYC binds SET minimal promoter region, interacting with and recruting SP1, RUNX1, GATA2 on SET promoter. Notably, modulation of MYC by genetic and/ or pharmacological approach reduced the levels of all the TFs above and, although it did not totally prevent the formation of the TFs complex, it abolished its localization on the SET functional promoter. This resulted in decreased SET expression at both mRNA and protein levels, and reduced cell proliferation rate *in vitro* and *in vivo* [83].

### Aberrant protein stability and cellular distribution

SET is mainly located within the nucleus of the cells (Figure 1A); however, in many forms of cancers and neurodegenerative diseases, SET translocates from its primarly nuclear localization to the cytoplasm, where it can form an inhibitory interaction with PP2A. As a nuclear protein, SET requires a specific sequence called the nuclear localization signal (NLS) to be targeted to the nucleus. The NLS of SET has been reported at 168KRSSQTQNKASRKR181, essential for the binding of SET with impα3, and targeted expression of SET in the cytoplasm is found to be associated with inhibition of PP2A activity and neuronal death, as discussed in more details in the following paragraph [95]. By using site-directed mutagenesis in combination with immunofluorescence analysis, 178RKR181 was identified as the minimal NLS sequence because mutations of these three aminoacidic residues caused SET to be diffusely expressed throughout the cytoplasm [96]. The shuttling of SET between nucleus and cytoplasm is also regulated by phosphorylation at Ser9 which inhibits the formation of the importin-α/importin-β complex required for the import of SET from the cytoplasm to the nucleus [30]. The Ser9 is nested in the center of the sequence [6] AKVSKK [11], which is also consistent with a classical NLS. Single aminoacid mutagenesis experiment of lysine 7, lysine 10, and lysine 11 to alanine acid (K7A, K10A, K11A) revealed that lysine 11 is also essential for the nuclear import of SET [30]. Phosphorylation on Ser9 also been described to increase the affinity between SET and PP2A [30]. This phosphorylation is mediated by the phosphoinositide-3-kinase γ (PI3Kγ) and Casein kinase 2 (CK2) and it has been correlated to aberrant cytosolic retention of SET in Alzheimer’s disease [30,31,97,98], in myeloproliferative neoplasms (MPNs) and AML [56,99]. In addition, other mechanisms including phosphorylation of SET on Serine 24 and 93 and sumoylation at Lysine 68 have also been described as critical sites to target SET in the cytosol; however, these modifications have only been described in models of Alzheimer’s disease [30,100]. Other mechanisms implicated in the cytosolic retention of SET are overexpression of SET binding protein 1 (SETBP1) [97,101] and interaction with Heterogeneous nuclear ribonucleoprotein A2 protein (hnRNPA2) (Table 1) [97,102]. Overexpression of SETBP1 protects SET from protease cleavage increasing the amount of full-length SET inside the cytoplasmic compartment with resultant SET-PP2A interaction and PP2A inactivation. Cristobal *et al.* showed that *SETBP1* was overexpressed in 53 of 192 AML patients (28%) and that SETBP1 overexpression correlated with poor overall survival [101]. More recently *SETBP1* has also been found mutated in myeloid leukemia and myelodisplastic syndrome, where it has been shown to regulate a transcriptional programme similar to those of *NPM1*-mutant and *KMT2A-*rearranged AMLs [103–106]. HnRNPA2 is a RNA and ssDNA-binding protein involved in different aspects of mRNA biogenesis and also described as a SET-binding protein and PP2A inhibitor. Although the specific mechanisms by which hnRNAP2 is involved in both SET stabilization and PP2A inhibition remain to be established, Vera *et al.* showed that SET and hnRNPA2 cooperate in PP2A inhibition [102].

### Aberrant Splicing

SET gives rise to four transcript variants: α, β, γ and δ [1,3]. These forms differ in the first exons and they have in common the sequences from exon 5 to exon 11. Among these, two SET isoforms, α and β, have been identified in CLL and NHL, with increased SETα isoform correlating with CLL disease severity [107,108]. This may suggest that alternative splicing of SET mRNA may be an important, yet unexplored oncogenic mechanism. Since phosphorylation on Ser9 of SET-β is responsible for the cytoplasmic accumulation of SET and PP2A inactivation and this phosphorylation is localised on the N-terminus of SET which distinguishes the α and β isoforms [30], it would be interesting to elucidate whether these two isoforms are equally localized within the subcellular compartments.

## The role of SET in cancer

As a phosphatase maintaining cell homoeostasis by counteracting the oncogenic kinase-driven signaling pathways, PP2A plays a fundamental role as tumor suppressor. Indeed, mutations in the genes *PPP2R1A* and *PPP2R1B* encoding for the scaffold subunit A, loss of the *PPP2CA* locus, encoding for the scaffold subunit C, and epigenetic silencing of genes encoding the regulatory B subunit (B55α, B56α, B56γ, B56δ) have been reported in several types of cancer [18,19,54,109]. However, genetic and epigenetic loss of PP2A function is relatively rare and it might not be the primary mechanism by which cancer cells inhibit PP2A activity. Functional mechanisms including post-translational modifications of catalytic and regulatory PP2A subunits and aberrant expression of PP2A endogenous inhibitors SET, SETBP1 and CIP2A represent novel emerging mechanisms for PP2A regulation in cancer [18,19,53,54,109]. By interfering with the PP2A-mediated dephosphorylation, SET activates oncogenic signaling such as AKT, ERK, c-MYC and GSK3-β to promote the survival and proliferation of cancer cells [18,19]. The oncogenic role of SET has been characterized in several cancer models by targeted genetic deletion achieved by RNA interference. Systematic analysis conducted by Kauko et al. in 2020, showed that SET has a profound effect on the phospho-proteomic profile of HeLa cells, regulating more than 30% of cellular phospho-peptides, with preference for chromatin associated nuclear proteins and CK2 targets [110]. Importantly, knockdown of SET sensitized cancer cells to anti-neoplastic treatment with staurosporin derivative and it enhanced phosphorylation in targets regulated by p53-protein associated complex and/or targets included within the PI3K/Akt/mTOR signaling pathway [110].

By using several models of BCR::ABL+ CML and B-ALL, Neviani *et al.* showed that the knockdown of SET and treatment with FTY720 results in re-activation of PP2A which, in turn, dephosphorylates and deactivates BCR/ABL via SHP1. As a result, the clonogenic potential trigged by BCR/ABL and SET was abolished both *in vitro* and* in vivo* [7,111]. Follow up studies from the same group revealed that SET-mediated PP2A inactivation was essential for the self-renewal of CML leukemic stem cells (LSCs) [94]. This has profound therapeutic implications, as we will discuss later. A marked reduction in PP2A activity was also found in CD34+ stem cells isolated from bone marrow and peripheral blood of JAK2-driven myeloproliferative neoplasms (MPN), compared with those isolated from healthy individuals [56]. Reactivation of PP2A by SET knockdown or pharmacological treatment with FTY720, increased survival in a mouse model of JAK2^V617F^ leukemia *in vivo* [56]. In NHL, expression of shRNA against SET resulted in a cytotoxic effect; these results highlight the importance of SET in NHL progression, indicating that SET overexpression is crucial for the proliferation of NHL cells [11]. In canine mammarian tumor cells lines of primary (CIP-p) and metastatic (CIP-m) origin, the role of SET overexpression was found specific for cancer progression at metastatic stage [112]. Indeed, SET knockdown suppressed cell anchorage and colony formation ability in CIP-m but not CIP-p and, at the metastatic stage, the antitumor effect of SET knockdown was found associated with PP2A re-activation and decreased levels of some of the main targets of PP2A, including phospho -GSK3-β and β-catenin. In addition to that, shRNA targeting SET (shSET) decreased the levels of cyclin D1, NFKβ, mTORC1 and p70S6Kinase. No effect was identified on on c-MYC, phospho-ERK, phospho-AKT and GSK3-β total protein. Moreover, only CIP-m cells were found sensititve to treatments with SET-PP2A targeting drugs, such as FTY720, which rescued PP2A activity, blocking cell proliferation at metastatic level [112]. Sobral *et al.* demonstrated *in vitro* and *in vivo* in head and neck squamous carcinoma (HNSCC) models, that knockdown of SET rescued the activity of PP2A, reduced the levels of phospho -ERK1/2 and phospho -p53(Ser15), impaired cell proliferation, enhanced cell sensitivity to the standard treatment with cisplatin, increased sensitivity to antineoplastic treatments and reduced tumour invasion [70]. The effect of SET on p53 stability was also reported in medulloblastoma. Wei *et al.* were the first to identify a signaling pathway mediated by SET-phospho-MDM2-p53 in p53-wild-type Sonic Hedgehog (SHH) medulloblastoma, a subgroup of medulloblastoma representing approximately 30% of all medulloblastoma cases and characterized by activation of SHH pathway. Whereas in physiological conditions MDM2 is dephosphorylated by PP2A on Ser166 residue, in SHH medulloblastoma the phosphorylated form of MDM2, which suppresses the activity of p53, was found overexpressed and accompanied by high levels of SET protein. Genetic modulation of SET by shRNA impaired cancer cell viability in a p53-dependent manner, indicating that SET plays a critical oncogenic role in the regulation of p53 in this tumor and it may represent an anti-neoplastic target for this disease [113]. Knockdown of SET increased c-MYC degradation and it decreased the tumorigenic potential of pancreatic cancer cell lines both *in vitro* and *in vivo* [92]. In gastric cancer (GC), where SET overexpression at both protein and mRNA levels represents a marker of poor prognosis, SET down-regulation by shRNAs impaired GC cell proliferation, colony formation, tumorigenesis, and metastasis *in vitro* and *in vivo* [91]. These effects were accompanied by reduction of cyclin D1, MMP2 (metalloproteinase2), c-MYC and phospho-AKT(Ser473) [91]. Further studies showed SET plays a crucial role for the stemness of gastric cancer cells [90]. One of the potential mechanisms involved in the maintenance of gastric tumorigenesis and stemness is represented by SET/PP2A/E2F1 axis. To note, E2F1 (E2F transcription factor 1) is responsible for the maintenance of cancer cells stemness and E2F1 stabilization is controlled by phosphorylation at Ser364 via PP2A. Microarray analysis showed that up-regulation of SET enriches the expression of E2F1, whilst knockdown of SET decreased the levels of this protein and suppressed colony formation in MKN45 and MKN75 human gastric cell lines [90]. In metastatic colon-rectal cancer, SET silencing by siRNA induced a decrease of cell growth and increased the sensitivity to the current oxaliplatin-based treatment [86]. In oral keratynocyte spontanously immortalized (NOK-SI) cell lines, accumulation of SET affects cellular signaling, morphology and cell metabolism, representing an initiating event for oncogenesis and tumor aggressiviness [114]. Analysis of targets commonly regulated by SET, such as AKT and ERK, showed that, in NOK-SI cells overexpressing SET (NOK-SI SET), the levels of phosphorylated AKT (Thr308), phosphorylated ERK (Thr 202 and Tyr204) and c-MYC proteins were higher than in the parental cell line and accompained by up-regulation of c-MYC and low expression of hyperphosphorylated Rb. Notably, in response to accumulation of SET, NOK-SI cells acquired unique stem-cell features compared with wild-type cells, such as lack of epithelial and mesenchymal stem-cell markers (CD71, integrin α6/β4, CD44 and CD73) [114]. Moreover, NOK-SI SET dysplayed higher levels of calcium and lipid droplets, increased mitochondrial number and density, higher ATP producion and glucose uptake associated with lower relase of lactate [114]. This piece of evidence implicates that overexpression of SET drives metabolism-reprogramming of NOK-SI cells and stimulates clonogenic potential *in vitro* and undifferentiated squamous cell carcinoma formation *in vivo* [114]. Recent studies have also indicated that through interaction with PP2A, SET upregulates androgen biosynthesis [34] and contributes to hyperandrogenism in polycystic ovary syndrome (PCOS) and to gynecologycal cancers, as reviewed by Jiang *et al*. [93,115].

**Figure 4:**
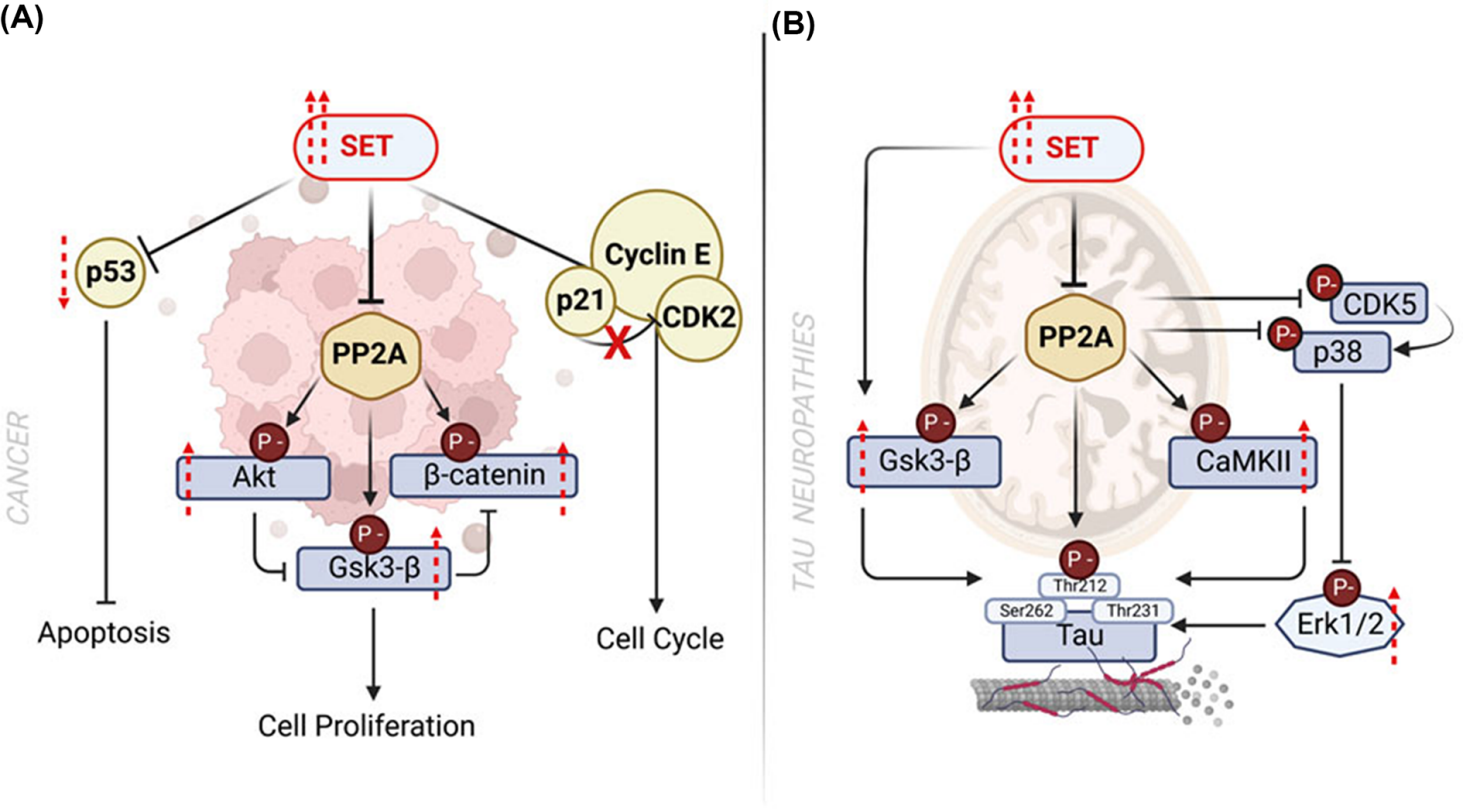
SET in cancer and tau neuropathies development Dysregulation of SET expression contributes to the development of cancer and tau neuropathies. (**A**) Overexpression of SET in cancer negatively impacts the activity of several tumor-suppressors such as p53, PP2A and the cyclin-dependent protein kinase complex (p21-Cyclin E-CDK2) leading to dysregulation of cell cycle checkpoints and uncontrolled proliferation of malignant cells. (**B**) In tau neuropathies, SET contributes to disease progression by inactivating the phosphatase activity of PP2A, the main tau phosphatase in the brain. PP2A regulates tau phosphorylation both directly and indirectly via several downstream targets such as GSK-3β and CaMKII, CDK5 and p38. Therefore, in response to PP2A inactivation, the Tau protein is hyperphosphorylated at several residues (Ser262, Thr212, Thr231) leading to accumulation of abnormal Tau aggregation on microtubules and loss of microtubule stability. Image generated using Biorender.

Overall, these data provide strong evidence that SET plays a crucial role in cancer via a mechanism involving PP2A inhibition (Figure 4A).

## SET in Alzheimer’s disease

SET has important implications in the pathogenesis of neurodegenerative tauopathies, including Alzheimer’s disease (AD) and amyotrophic lateral sclerosis (ALS). These diseases are characterized by extracellular deposits of Aβ, the proteolytic by-product of the transmembrane protein APP and by an abnormal hyperphosphorylation and aggregation of microtubule-associated protein tau into paired helical filaments/neurofibrillary tangles, causing its aggregation, breakdown of the microtubule network, impairment in microtubule dynamics, axonal transport, and neurite outgrowth, which synergistically contribute to the pathogenic process and eventually cell death. The phosphorylation status of tau is tightly regulated by a balance between protein kinases and phosphatases. The tau kinases are divided in three groups: proline-dependent protein kinases (PDPKs), non-PDPKs, and tyrosine protein kinases (TPK) [116]. Approximately 50% of the tau phosphorylation sites are canonical (Ser/Pro,Thr/Pro) for PDPKs and the remaining sites are phosphorylated by non-PDPKs. In addition, TPK can phosphorylate tau at 5 tyrosine residues [116]. PP2A is the major brain tau phosphatase accounting for over 70% of tau phosphatase activity in the human brain [20,117–119]. PP2A regulates tau phosphorylation both directly and indirectly by modulating the activity of some PDPKs including glycogen synthase kinase-3β (GSK-3β), cyclin-dependent kinase 5 (cdk5), p38 MAPK and extracellular signal-regulated kinases 1 and 2 (ERK1/2) and non PDPKs such as Ca^2+^/calmodulin-dependent protein kinase II (CaMKII), PKB/AKT [20,116–120]. Therefore, mechanisms implicated with the activity of PP2A are likely to affect tau. Indeed, several abnormalities of PP2A have been reported in AD, including decreased mRNA and protein levels of the PP2A subunit C, decreased protein levels of the PP2A subunits A and B (B55α, B55γ, B56ε), reduced PP2A C methylation at Leu309 by leucine carboxyl methyltransferase 1 (LCMT1), down-regulation of expression of LCMT1, increased cleavage of PP2A regulator α4, and overexpression of SET and ANP32A [117,118]. The link between SET and AD became apparent when SET expression was found increased in the hippocampus of AD patients [4–6]. Tsujio *et al*. reported the expression of SET in human brains and they demonstrated that, in this context, SET inhibited PP2A activity resulting in hyperphosphorylated tau and cell death [5]. The same year, Madeira *et al.* showed that expression of SET induces cell death in primary neurons and that SET interacted with APP [64]. This study provided a first evidence of the cytotoxic effect of SET in neurons. A further confirmation of this mechanism was provided by *in vivo* experiments of Wang *et al*. [121]. The authors reported that forced expression of SET in the brain of rats decreased PP2A activity, and it induced abnormal hyperphosphorylation of tau, neurodegeneration, spatial reference memory and memory consolidation, deficits typical of AD [121]. Interestingly, the authors found that the inactivated form of GS3Kβ, phosphorylated on Ser9, (pSer-9-GS3Kβ) was decreased upon SET expression. Therefore, SET contributes to amyloidogenesis and to an abnormal hyperphosphorylation of tau by a dual mechanism, via inhibition of PP2A and activation of tau kinase GS3Kβ. Whereas Wang et al. did not report any effect on total level of GS3Kβ upon SET expression, Zhang et al. showed that knockdown of SET inhibited GS3Kβ activity by decreasing the levels of GSK3β mRNA and protein with an increased inhibitory phosphorylation of GS3Kβ at Ser9 in HEK293/tau and N2a/tau cells, and human tau (htau) transgenic mice [98]. This suggests that SET might regulate the activity of GS3Kβ by modulating its expression. Interestingly, GSK3β can also regulate the expression of SET [122]. This establishes a feedback-loop mechanism whereby expression of SET inhibits PP2A which results in hyperphosphorylation of tau and iper-activation of tau kinases GSK3β which can sustain SET-overexpression by regulating SET RNA stability and translation [121,122].

Likewise, forced expression of SET by adeno-associated vectors in the brain of newborn rats, produced reference memory impairment, tau pathology and intraneuronal accumulation of Aβ by 5–8 months. Moreover, by the age of 10**–**14 months the rats showed motor deficit and hyperphosphorylation and proliferation of neurofilaments and tau [123]. In neuronal cell lines, expression of SET results in abnormal hyperphosphorylation of tau at Thr231/Ser235, Ser262/356, and Ser396/404, which are sites known to inhibit binding of tau to microtubules [29]. Interestingly, in the hippocampus of the temporal cortex of AD patients, SET was found accumulated in the cytosol and it co-localized with phosphorylated tau and cleaved amyloid precursor protein (APPcc) [6,124], suggesting that specific accumulation of SET in the cytosol could contribute to the pathogenesis of AD. This was proved by experiments performed by Qu *et al.* that, by using deletion mutants, showed that under genotoxic stress, targeted expression of SET to the cytoplasm induces cell death in cortical neurons [95]. In addition, studies by Arif *et al*. and Chasseigneaux *et al.* show that forced expression of SET in the cytoplasm of neuronal cells is sufficient to inhibit PP2A activity and activate tau kinases, including GSK3β and CAMKII, leading to tau hyperphosphorylation [96,125]. As discussed above, the translocation of SET between nucleus and cytoplasm is mostly regulated by phosphorylation at Ser9 [30] and it is mediated by PI3Kγ and CK2 [30,31,98]. A recent study has demonstrated that Aβ or tau overexpression in primary neuronal cultures can induce CK2 activation and SET Ser9 phosphorylation, resulting in its cytoplasmic translocation [98]. More importantly injection of SET Ser9 phosphorylation mimetic mutant was sufficient to induce tau pathology and behavior impairments in AD mouse models, providing evidence of the pathogenetic role of SET Ser9 phosphorylation [98]. Overall these data suggest that SET plays a crucial role in AD neurodegeneration via mechanisms involving PP2A inhibition (Figure 4B).

## SET as a new target for anticancer and anti-Alzheimer therapies

Several studies have indicated that PP2A inactivation is a key molecular pathogenetic event in cancer and AD and that pharmacological strategies to re-activate PP2A might unfold new therapeutic opportunities, as reviewed [18,53,54,109,118,119,126–132]. Given the role of SET in PP2A inactivation and downstream pathways involved in oncogenic signaling and converging on hyperphosphorylation of tau, SET could represent a valuable target for treatment of cancer and AD. Several studies have reported the therapeutic effects of FTY720 in distinct cancer models, as reviewed [56,94,111]. Despite the anti-tumor effects, FTY720 elicits severe cardiac toxicity, among other side effects, limiting its applicability in clinical settings. In addition to FTY720, novel specific SET-antagonist peptides such as OP499 and COG112 are currently under development for cancer therapy [53,131]. These antagonists suppress the activity of SET by a direct interaction with SET, which results in increased PP2A activity and anticancer effect. This paradigm has been demonstrated in hematological malignancies including CML, AML and NHL [11,82], as well as in several models of solid tumors [69,90,92,113]. Notably, as SET-mediated PP2A inactivation is essential for the self-renewal of CML LSCs [94], re-activation of PP2A could represent a strategy to eradicate quiescent LSCs, that are typically refractory to kinase inhibitors such as imatinib [53,94,133]. Therefore, more studies are needed to evaluate the specific effect of SET inhibition on LSCs as well as on cancer stem cells (CSCs) isolated from solid tumors.

Interestingly, a recent study has reported that PP2A inactivation contributes to epigenetic silencing of MLH1, resulting in MMR defects and MSI [51]. As MMR defects lead to accumulation of mutations that can be a source of immunogenic neoantigens, these deficiencies could be synthetic lethal with immune checkpoint blockade (ICB), the cornerstone of immunotherapy-based anticancer strategies for solid tumors. More studies are needed to dissect the specific role of SET in MMR and to identify those tumors that could benefit from ICB.

The therapeutic potential of anti-SET based strategies for AD has been highlighted by studies conducted in mouse models. Silencing of SET by RNA interference attenuated amyloidogenesis and improved learning and memory in Tg2576 mice, a mouse model overexpressing a mutant form of APP, linked to early-onset familial AD [134]. The study indicated that silencing SET, reduced the accumulation of Aβ in the hippocampus and cortex of the AD mice by reducing APP phosphorylation and inhibiting β-secretase with a mechanism dependent on activation of PP2A [134]. Notably, the authors did not report a prominent effect on tau dephosphorylation in this model. To specifically investigate the effect of SET silencing on tau pathologies, the same group employed a human tau (htau) transgenic mouse [98]. In this model, SET genetic silencing led to reduction of tau phosphorylation/accumulation and improvement of memory deficits [98]. Notably, in addition to the increase in PP2A activity, SET silencing led to inhibition of GSK3β [98]. As both PP2A and GSK3β regulate phosphorylation of tau, silencing SET might reduce tau phosphorylation by two complementary mechanisms, activation of de-phosphorylation and inhibition of phosphorylation. Although these mouse models present a beautiful proof of concept that silencing SET contrasts the pathogenesis of AD, we are still far from being able to directly edit the genome in human patients. Theoretically, most compounds that modulate PP2A activity [119,132] may affect the tau phosphorylation and Aβ generation, however whether FTY720 and OP499 exhibit these effects has not been elucidated. COG112, an apolipoprotein E mimetic, inhibits the interaction between SET and PP2A with resulting increased PP2A activity and reduction in phosphorylation of tau in the hippocampus and cerebral cortex of AD mouse models [135].

As Ser9 phosphorylation triggers not only SET translocation to the cytosol but it also increases the affinity between SET and PP2A [30], strategies to target this phosphorylation might provide a novel therapeutic approach for cancer and AD [98]. A recent study shows that CK2 pharmacological inhibition reduces the accumulation of tau *in vitro*, but whether these effects are mediated by SET exclusion from the cytosol and whether this inhibition would ameliorate AD symptoms *in vivo* is not known [136]. In AML models, CK2 genetic silencing and pharmacological inhibition did not result in exclusion of SET from the cytosol of AML cells, indicating that targeting a single kinase might not be sufficient to release PP2A from SET inhibition [99]. Therefore, studies addressing the specific effects of SET silencing and SET antagonists in relevant disease models are needed.

## Conclusions

SET is a protein with multiple functions ranging from regulation of gene expression, cell migration, survival and differentiation which has been implicated in the progression of solid and haematological malignancies as well as in tauopathies such as AD. Multiple mechanisms including aberrant gene expression, splicing and cellular localization have been shown to contribute to aberrant activity of SET in cancer and in AD. Whereas for AD there is abundance of evidence pointing to a pathogenetic role of SET associated to its inhibitory effect on PP2A and on the downstream hyperphosphorylation of tau, it is not completely understood whether PP2A inhibition could explain the plethora of oncogenic effects mediated by SET or if other SET functions, including inhibition of acetyltransferase or binding to specific partners, contribute to cancer progression and development of drug resistance. Given that SET is both a member of the INHAT complex and an endogenous inhibitor of PP2A, it would be interesting to explore whether the dual modulation of histone phosphorylation and acetylation contribute to the pathogenic effects of SET. Several studies, have indicated that SET-binding peptides such as COG112 and OP449 inhibit the interplay between SET and PP2A, block malignant progression and reduce phosphorylation of tau in AD mouse models, providing a proof of concept that targeting SET might represent a new therapeutic approach for cancer and AD. Efforts to understand the exact contribution of SET in specific tumors and healthy tissues might, therefore, pave the way to the development of novel specific drugs for treatment of cancer and AD.

## Abbreviations

AD: Alzheimer’s disease
CaMKII: calmodulin-dependent protein kinase II
cdk5: cyclin-dependent kinase 5
CSC: cancer stem cell
ERK1/2: extracellular signal-regulated kinases 1
GSK-3β: glycogen synthase kinase-3β
HNSCC: head and neck squamous carcinoma
ICB: immune checkpoint blockade
LCMT1: leucine carboxyl methyltransferase 1
SHH: Sonic Hedgehog
